# Characterization of the rice NLA family reveals a key role for OsNLA1 in phosphate homeostasis

**DOI:** 10.1186/s12284-017-0193-y

**Published:** 2017-12-28

**Authors:** Jian Yang, Lan Wang, Chuanzao Mao, Honghui Lin

**Affiliations:** 1 Ministry of Education Key Laboratory for Bio-Resource and Eco-Environment, College of Life Science, Sichuan University, Chengdu, 610064 China; 2 Biogas Institute of Ministry of Agriculture, Chengdu, 610041 China; 3 State Key Laboratory of Plant Physiology and Biochemistry, College of Life Sciences, Zhejiang University, Hangzhou, 310058 China

**Keywords:** Rice, Phosphate, OsNLA1, Pi-homeostasis

## Abstract

**Background:**

Phosphate (Pi), an essential mineral nutrient for plant development and reproduction, is one of the main components of fertilizers in modern agriculture. Previous research demonstrated that AtNLA1 mediates ubiquitination of Pi transporters in the plasma membrane and triggers their endocytosis and degradation in Arabidopsis. In this study, we researched the function of NLA homologous proteins in Pi homeostasis in rice.

**Findings:**

Two *OsNLA* homologs from rice (*Oryza sativa* L.) were identified by bioinformatics and phylogenetic analysis and designated *OsNLA1* and *OsNLA2*. The *OsNLA1* clustered with Arabidopsis *AtNLA1*, was expressed higher than *OsNLA2* and was transcriptionally repressed under Pi-deficient condition. Loss-of-function of *OsNLA1* caused P overaccumulation and growth inhibitions in both root and shoot under Pi-sufficient condition. Furthermore, mutation of *OsNLA1* affected expression of Pi tranporters and root hair development under Pi-sufficient and/or Pi-deficient conditions.

**Conclusions:**

*OsNLA1* plays a key role in maintaining phosphate homeostasis in rice.

**Electronic supplementary material:**

The online version of this article (10.1186/s12284-017-0193-y) contains supplementary material, which is available to authorized users.

## Findings

Phosphorus (P) is a mineral nutrient essential for plant development and reproduction, and is integral to several macromolecules such as phospholipids and nucleic acids. Despite the indispensable role of P for plants, levels of phosphate (orthophosphate; Pi), the only form of P that can be taken up by plants, are commonly limited because of chemical fixation and microbial activity (Raghothama, 1999). To cope with suboptimal Pi conditions, plants have developed a series of adaptive responses, such as induction of Pi transporters and modification of root system architecture (Raghothama, 1999; Lin et al., 2009; Wu et al., 2013). Plant uptake of Pi is largely mediated by plasma membrane -localized Pi transporters belonging to the PHOSPHATE TRANSPORTER1 (PT) symporter family. Thirteen *PT* genes have been identified in rice (*Oryza sativa*) and nine in *Arabidopsis thaliana* (Goff et al., 2002; Karthikeyan et al., 2002). *OsPTs* differ in tissue expression patterns and affinities for Pi, resulting in diverse functions in plants. For instance, the high-affinity Pi transporter *OsPT8* is universally expressed in rice, and is responsible for half of its Pi uptake (Chen et al., 2011; Jia et al., 2011). Although most *OsPTs* in rice are induced at the transcriptional level by Pi starvation or mycorrhizal symbiosis (Yang et al., 2012; Secco et al., 2013), post-transcriptional regulating of OsPT family proteins is also important to their activities (Gonzalez et al., 2005; Bayle et al., 2011; Chen et al., 2011; Chen et al., 2015). NITROGEN LIMITATION ADAPTATION (NLA), designated AtNLA1 in this study, was first identified as a positive regulator for the adaptability of Arabidopsis to nitrogen limitation (Peng et al., 2007), and later analysis of Pi concentration revealed that the early senescence phenotype of *atnla* mutant plants was due to Pi toxicity (Kant et al., 2011). In Arabidopsis, AtNLA1 can interact with AtPTs members via its SPX domain, and mediate ubiquitination of AtPTs in the plasma membrane and trigger their endocytosis and degradation (Lin et al., 2013; Park et al., 2014). Recently, two research groups separately reported roles of *OsNLA1* in mantaining Pi homeostasis in rice (Yue et al., 2017; Zhong et al., 2017). Yue et al., (2017) additionally reported OsNLA1 functioned as a ubiquitin ligase to degrade Pi transporters in rice, with a similar function of AtNLA1 in Arabidopsis.. In this study, we were interested in the phylogenetic relationship of the NLA family and expression of *OsPTs* and root hair development in *osnla1* mutant.

**Fig. 1:**
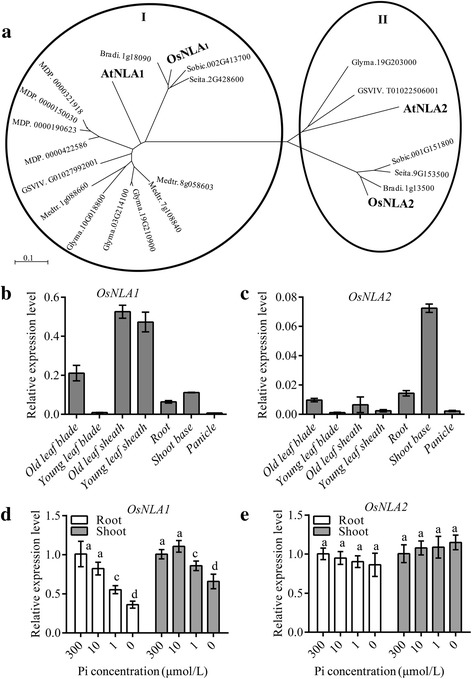
Phylogenetic relationships of NLA proteins and *OsNLA* family expression patterns in rice. **a** Unrooted phylogenetic tree of the NLA family using MEGA 5.10 by the neighbor-joining method. Dicots: *Vitis vinifera* (GSVIV), *Glycine max* (Glyma), *Malus domestica* (MDP), *Medicago truncatula* (Medtr), *Arabidopsis thaliana* (At); monocots: *Brachypodium distachyon* (Bradi), *Sorghum bicolor* (Sobic) and *Setaria italica* (Si). *OsNLA* family expression patterns in rice. **b**, **c** Spatial expression of *OsNLAs* transcripts. Transcript levels for *OsNLA1* (b) and *OsNLA2* (c)in leaf sheath, leaf blade, roots and shoot base in 30-d-old seedlings grown in nutrient solutions containing 300 μM Pi and in panicle before flowering. Expression of *OsNLA1* and *OsNLA2* is relative to *OsACTIN2*. **d**, **e** Transcript levels of *OsNLA1* (d) and *OsNLA2* (e) in root and shoot of plants grown for 10 d in nutrient solutions with different Pi concentrations. Data represent mean ± SD of three replicates. Different letters represent significant differences according to Duncan’s multiple range test (*P* < 0.05)

An unrooted phylogenetic analysis of the NLA family proteins with four monocots (*B. distachyon*, *S. bicolor*, *S. italica* and rice) and five dicots (grapevine, soybean, apple, *M. truncatula* and Arabidopsis), revealed the presence of two distinct clades. Although all plants had proteins belonging to clade I, in which AtNLA1 involved in regulating Pi homeostasis was present (Kant et al., 2011), all monocots and only some dicots had NLA members belonging to clade II (Fig. 1a). This suggested that NLA members of clade I conservatively functioned in maintaining Pi homeostasis among different plant species. Quantitative reverse-transcription PCR (qRT-PCR) was performed on different tissues for rice plants grown in nutrient solutions under Pi- sufficient (300 μM) condition (Additional file 1). The amplification efficiencies of gene-specific primers for *OsNLA1* and *OsNLA2* were assessed and found that they were approximately equal (95.5% for *OsNLA1* and 94.3% for *OsNLA2*) (Additional file 2: Figure S1 and Additional file 3: Table S1). Then, the transcript level of *OsNLA1* and *OsNLA2* in plants were compared, and found that the transcript level of *OsNLA1* was higher than that of *OsNLA2* in all tissues tested, with about 1.5-fold in shoot base, 4.5-fold in root and 80-fold in leaf sheath (Fig. 1b and c). Previous transcriptome analysis also shown that *OsNLA1* abounace was higher than *OsNLA2* in both root and shoot (Secco et al., 2013). Furthermore, the *OsNLA1* transcript was differentially regulated by Pi availability, with higher expression in Pi- sufficient and lower expression in Pi -deficient conditions (Fig. 1d), as *AtNLA1* in Arabidopsis (Lin et al., 2013); however, the *OsNLA2* transcripts remained at relatively constant levels (Fig. 1e). The transcriptional change of *OsNLA1* in response to Pi supply was also identified by RNA sequencing (Secco et al., 2013). Based on phylogenetic relationships and expression levels and responses to Pi starvation of the *NLA* family in rice, we suggest that *OsNLA1* might plays a major role in regulating Pi homeostasis, as does *AtNLA1* in Arabidopsis.

**Fig. 2:**
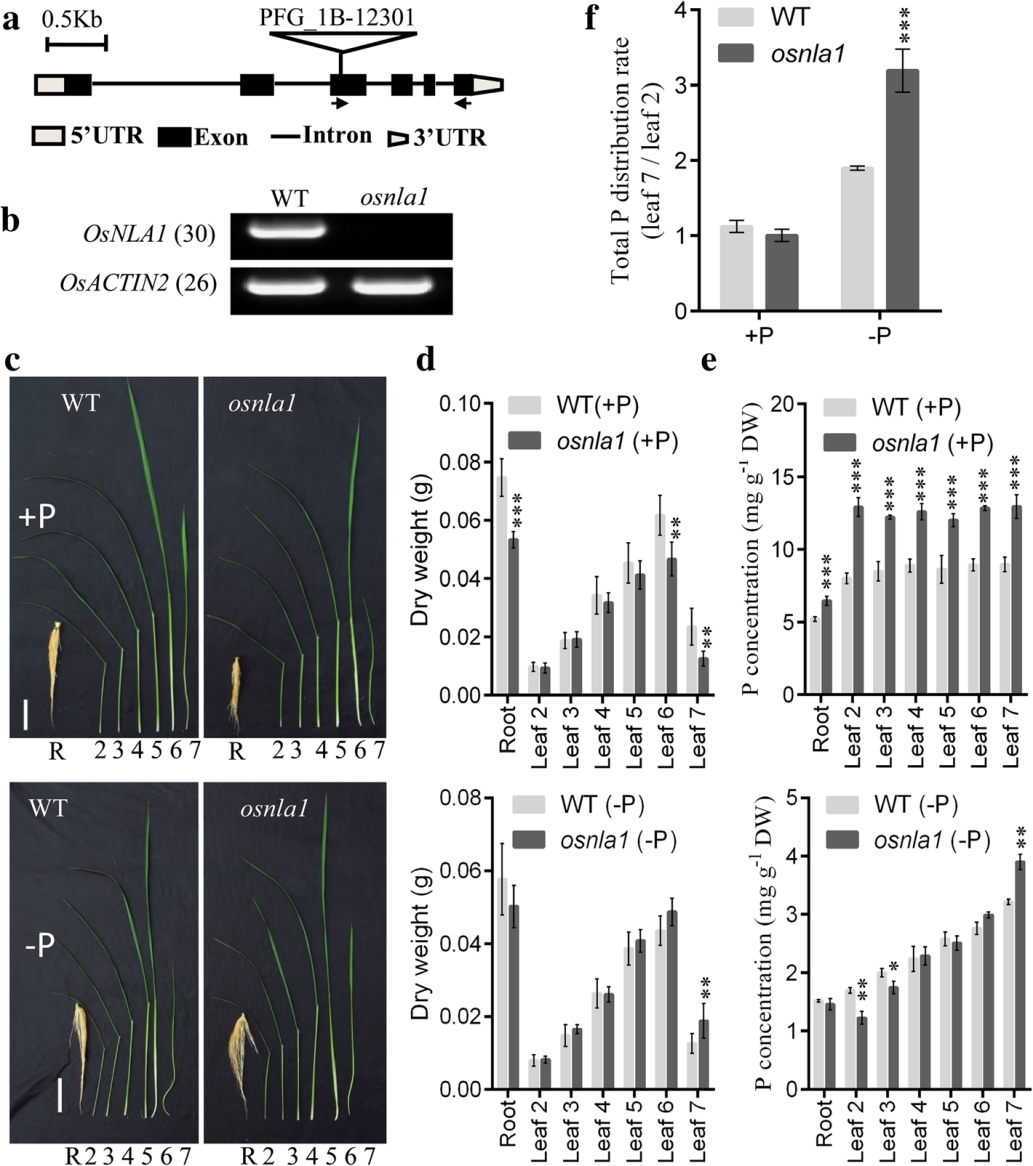
Phenotypical characteristics of *osnla1* mutant. **a** Genomic structure of rice *OsNLA1*. Position of the T-DNA insertion in *OsNLA1* is indicated by a triangle. Small arrows are the gene-specific primers for RT-PCR. **b** RT-PCR analysis of *OsNLA1* expression in roots of the mutant and wild-type (Dongjin; WT). **c** Phenotype comparison between WT and *osnla1* mutant. 30-d-old WT and *osnla1* grown under Pi-sufficient (300 μM; +P; upper) and Pi-deficient (10 μM; -P; lower) conditions. Bars = 5 cm*.* **d** Biomass of 30-d-old WT and *osnla1* in (**c**). Data represent mean ± SD of eight replicates. **e** Total P concentration in different leaves and roots of 30-d-old WT and *osnla1* in (c). Data represent mean ± SD of three replicates. **f** Distribution ratio of total P between young leaves (leaf 7) and old leaves (leaf 2) in *osnla1* and WT. Asterisks represent a significant difference with the corresponding WT (**, *P* < 0.01; ***, *P* < 0.001)

To characterize the functions of the *NLA* gene family in rice, we searched for publicly available mutants in different rice genomic resources. One T-DNA null mutant in *OsNLA1* gene (PFG_1B-12,301) was obtained from RISD DB (Rice T-DNA Insertion Sequence Database) (Fig. 2a and b). After growth for 30 d under Pi-sufficient condition (300 μM Pi; +P), the shoots and roots of *osnla1* were inhibited compared with wild-type (WT) plants (Fig. 2c and d). In addition, *osnla1* displayed leaf tip necrosis on old leaves, which was a typical Pi toxicity symptom in rice (Fig. 2c, Additional file 2: Figure S1). This symptom in *osnla1* was not observed when grown in Pi-deficient condition (10 μM Pi; -P). Moreover, the inhibited root phenotype of *Osnla1* was reversed when grown in Pi-deficient condition (Fig. 2c and d). Total P concentrations in all tissues of *osnla1* were higher than of WT, with 1.24-fold in roots and 1.46-fold in leaves under Pi-sufficient condition (Fig. 2e). This indicating that *OsNLA1* played a key role in Pi uptake in rice, as previously reported (Yue et al., 2017; Zhong et al., 2017)*.* However, under Pi-deficient condition, total P concentrations in old leaves (leaves 2 and 3) of *osnla1* were decreased by 17–25%, while total P concentrations in youngest leaves (leaves 7) of *osnla1* were increased by 21% compared with WT. The total P distribution rate in WT plants grown in Pi-deficient condition was 1.68-fold higher than that in plants grown in Pi-sufficient condition. However, the rate in *osnla1* mutants grown in Pi-deficient condition was 3.19-fold higher than that in plants grown in Pi-sufficient condition (Fig. 2f). This significant increased total P distribution rate under Pi limiting condition sustained the newly leaves growth (Fig. 2c and d). Pi is the major form of P transported within the plants, and old leaves Pi pool would be the source of Pi in regard to young leaves under Pi-deficient condition, resulting in higher P concentration in young leaves (Li et al., 2015, b). Thus, these results indicated that *OsNLA1* was also involved in Pi remobilization besides Pi uptake. This was expected because *OsPT1* and *OsPT8* also function in the redistribution of Pi from source to sink organs (Sun et al., 2012; Li et al., 2015, b). In a recent study, AtNLA1 was also involved in mediating degradation of NRT1.7 and further remobilizing nitrate from source to sink in Arabidopsis (Liu et al., 2016). Whether *OsNLA1* also functions in nitrate remobilization need further studies.

**Fig. 3:**
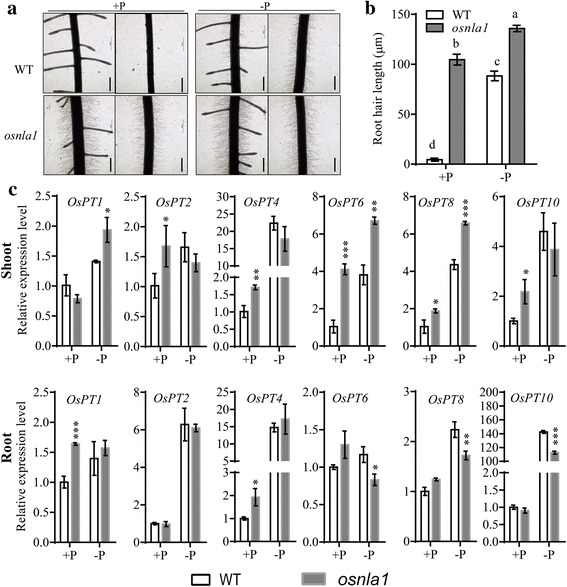
Root hair proliferation and expression of Pi transporter genes in *osnla1* mutant and wild-type (WT). **a** Root hair proliferation of WT and *osnla1* grown on Pi-sufficient (+P; left) and Pi-deficient (–P; right). Bars = 100 μm. **b** Root hair length in the maturation zone of roots. Data represent mean ± SD of eight replicates. Different letters represent significant differences according to Duncan’s multiple range test (*P* < 0.05). **c** Expression of Pi transporter genes in *osnla1* mutant and WT. Ten-day-old plants grown in Pi-sufficient (300 μM Pi) nutrient solution were transferred to Pi-sufficient (+P) and -deficient (-P) conditions for 10 d. RNA was extracted from shoots (upper) and roots (lower) for qRT-PCR. Data represent mean ± SD of three replicates. Asterisks represent significant difference with the corresponding WT (*, *P* < 0.05; **, *P* < 0.01; ***, *P* < 0.001)

As plants may modify root system architecture when growth in suboptimal Pi condition, we analyzed root hairs when *osnla1* and WT were grown in Pi-sufficient and -deficient conditions. After Pi-deficient growth for 10 d, WT developed many root hairs (Fig. 3a and b), as previously reported (Zhou et al., 2008; Sun et al., 2012). However, the length of root hairs on *osnla1* mutant was 1.5-fold those of WT plants grown in Pi-deficient condition. Furthermore, *osnla1* mutant also had increased root hairs length under Pi-sufficient condition compared with WT plants. Since OsNLA1 could mediate the degradation of OsPT2 and OsPT8 (Yue et al., 2017), inhibition of root growth and induce of root hair in *osnla1* was expected because *OsPTs* were involved in regulating root growth and root hair development (Jia et al., 2011; Sun et al., 2012).

Since changing the expression of *OsPT4* or *OsPT8* affects the expression of other Pi transporters in rice (Jia et al., 2011; Sun et al., 2012; Li et al., 2015, b) and protein levels of OsPT2 and OsPT8 accumulated in *osnla1* mutants (Yue et al., 2017), we then analyzed the transcriptional levels of *OsPTs* following WT and *osnla1* mutant growth in Pi-sufficient and -deficient conditions for 10d. In the shoot of *osnla1* mutant, transcripts of most of Pi transporters were induced (Fig. 3c). Compared with the WT, expressions of *OsPT6* and *OsPT8* were greatly induced under both Pi-sufficient and -deficient conditions. Although *OsPT2*, *OsPT4* and *OsPT10* were also up-regulated under Pi-sufficient condition, their transcript levels did not change under Pi-deficient condition. Expression of *OsPT1* was induced only when *osnla1* mutant was grown under Pi-deficient condition. However, contrary to our finding, Yue et al. (2017) found that *OsPT2* and *OsPT8* were unchanged in leaf under Pi-sufficient conditions. This might be resulted from transcriptional levels of *OsNLA1* and Pi transporters differed in various tissues (Fig. 1b; Remy et al., 2012). Unlike Pi transporters induced in the shoot, transcripts of Pi transporters were differentially regulated under Pi-sufficient and -deficient conditions in root of *osnla1* mutant (Fig. 3c). The transcriptional levels of *OsPT1* and *OsPT4* were induced under Pi-sufficient condition, but unchanged under Pi-deficient condition. In contrast, *OsPT6*, *OsPT8* and *OsPT10* were down-regulated under Pi-deficient condition, but unchanged under Pi-sufficient condition. The increased or repressed expression of these Pi transporters was caused, at least in part, by accumulated protein level of Pi transporters in *osnla1* mutant, because changing the expression of *OsPT4* or *OsPT8* affects the expression of Pi transporters in rice (Jia et al., 2011; Zhang et al., 2015). Moreover, induced expression of *OsPT1* and *OsPT8* in shoot of *osnla1* mutant under Pi-deficient condition would further remobilize Pi from old to young leaves (Sun et al., 2012; Li et al., 2015, b).

Since OsNLA1 mediates degradation of OsPTs and plays a key role in maintaining Pi homeostasis in rice. In this research, we identified OsNLA1 could regulate root system architecture, Pi transporters at the transcriptional levels and Pi redistribution from source to sink organs. These results presented here will provide a novel insight into the function of OsNLA1 in rice.

## Additional files

Additional file 1: Materials and methods. (DOCX 19 kb)

Additional file 2: Figure S1. Calculation of PCR efficiencies. **Figure S2.** Leaf blades of 30-d-old WT and *osnla1* grown under Pi-sufficient (300 μM; +P) and Pi-deficient (10 μM; -P) conditions. (PPTX 304 kb)

Additional file 3: Table S1. Primers used in this study. (DOCX 14 kb)

## Abbreviations

NLA: Nitrogen Limitation Adaptation
Pi: Phosphate
PT: Phosphate transporter
RISD DB: Rice T-DNA Insertion Sequence Database
WT: Wild type
