# Supplementary material for: Characterization of the rice NLA family reveals a key role for OsNLA1 in phosphate homeostasis
Source: Rice (N Y). 2017 Dec 28;10:52. doi: 10.1186/s12284-017-0193-y (PMC5745205; doi:10.1186/s12284-017-0193-y)
Supplement: Supplementary file 1 — Materials and methods. (DOCX 19 kb) [file 12284_2017_193_MOESM1_ESM.docx]

# Materials and Methods

# Plant materials and growth conditions

The *Osnla1* mutant (PFG_1B-12301) and its WT (Dongjin) were obtained from RISD DB (http://cbi.khu.ac.kr/RISD_DB.html). Rice seeds were sterilized for 30 min with diluted 30% NaClO, followed by thorough rinsing for 30 min with deionized water. Seeds were germinated in darkness at 28 °C for 3 d. Rice plants were cultured in nutrient solution (Yoshida et al., 1976) and grown in a green house with a 12/12-h light/dark cycle at 30/22 °C, approximately 200 mmol m^–2^s^–1^ photon density and approximately 60% relative humidity ([Yang et al., 2016](#_ENREF_2)).The solution was adjusted to pH 5.5 with 1 M NaOH or 1M HCl before use, and renewed every 3 d.

## Phylogenetic analysis

Amino acid sequences for putative NLA proteins were retrieved from Phytozome (https://phytozome.jgi.doe.gov/) and the Arabidopsis Information Resource (http://www.arabidopsis.org/). An unrooted phylogenetic tree was constructed using MEGA 5.10 by the neighbor-joining method with parameters of pairwise deletion, Poisson correction model of amino acid substitutions and 1000 bootstrap replications as previously described ([Yang et al., 2016](#_ENREF_2)). Protein sequence data can be found in databases under thefollowing accession numbers: GSVIVG01027992001, GSVIVT01022506001, Glyma.19G210900, Glyma.19G203000, Glyma.10G018800, Glyma.03G214100, Medtr8g058603, Medtr7g108840, Medtr1g088660, MDP0000422586, MDP0000150030, MDP0000321918, MDP0000190623, Bradi1g18090, Bradi1g13500, Sobic.002G413700, Sobic.001G151800, Seita.2G428600, Seita.9G153500, AT1G02860 (AtNLA1), AT2G38920 (AtNLA2), LOC_Os07g47590 (OsNLA1) and LOC_Os03g44810 (OsNLA2).

## RNA extraction, RT-PCR and qRT-PCR

Total RNA from rice was extracted with a RNA extraction kit (NucleoSpin® RNA Plant, MACHEREY-NAGEL). First-strand cDNAs were synthesized from total RNA using SuperScript III reverse transcriptase (Invitrogen). RT-PCR was performed using a pair of gene-specific primers. qRT-PCR was performed using a TransStart Green qPCR SuperMix kit (Beijing TransGen Biotech; http://www.transbionovo.com/), according to the manufacturer’s instructions, and SYBR Green detection. Triplicate quantitative assays were performed on each cDNA sample. Amplification efficiencies were calculated according to the equation E = 10^(-1/slope)^. The relative expression was calculated using 2^−ΔΔCt^ Method ([Livak and Schmittgen, 2001](#_ENREF_1)). ΔΔCt = (*C*t_Target gene_ - *C*t*_OsACTIN2_*)_treatment_ - (*C*t_Target gene_ - *C*t*_OsACTIN2_*)_control_. *OsACTIN2* was selected as the internal control gene for RT-PCR and qRT-PCR. Primers for RT-PCR and qRT-PCR analyses are listed in Table S1.

## Analysis of total P concentrations

Dry tissues (~100 mg) of *osnla1* mutant and WT were digested with H_2_SO_4_ and H_2_O_2_ at 280 °C, and then P concentrations were determined using a continuous flow analyzer (SKALAR, SKALAR San plus system), as described previously (Chen et al., 2011).

**References**

Chen J, Liu Y, Ni J, Wang YF, Bai YH, Shi J, Gan J, Wu ZC, Wu P (2011) OsPHF1 regulates the plasma membrane localization of low- and high-affinity inorganic phosphate transporters and determines inorganic phosphate uptake and translocation in rice. Plant Physiol 157: 269-278

Livak KJ, Schmittgen TD (2001) Analysis of relative gene expression data using real-time quantitative PCR and the 2^−ΔΔCt^ method. Methods 25: 402-408

Yang J, Gao MX, Hu H, Ding XM, Lin HW, Wang L, Xu JM, Mao CZ, Zhao FJ, Wu ZC (2016) *OsCLT1*, a CRT-like transporter 1, is required for glutathione homeostasis and arsenic tolerance in rice. New Phytol 211: 658-670
