# Supplementary material for: Characterization of the rice NLA family reveals a key role for OsNLA1 in phosphate homeostasis
Source: Rice (N Y). 2017 Dec 28;10:52. doi: 10.1186/s12284-017-0193-y (PMC5745205; doi:10.1186/s12284-017-0193-y)
Supplement: Supplementary file 2 — Calculation of PCR efficiencies. Figure S2. Leaf blades of 30-d-old WT and osnla1 grown under Pi-sufficient (300 μM; +P) and Pi-deficient (10 μM; -P) conditions. (PPTX 304 kb) [file 12284_2017_193_MOESM2_ESM.pptx]

## Slide 1
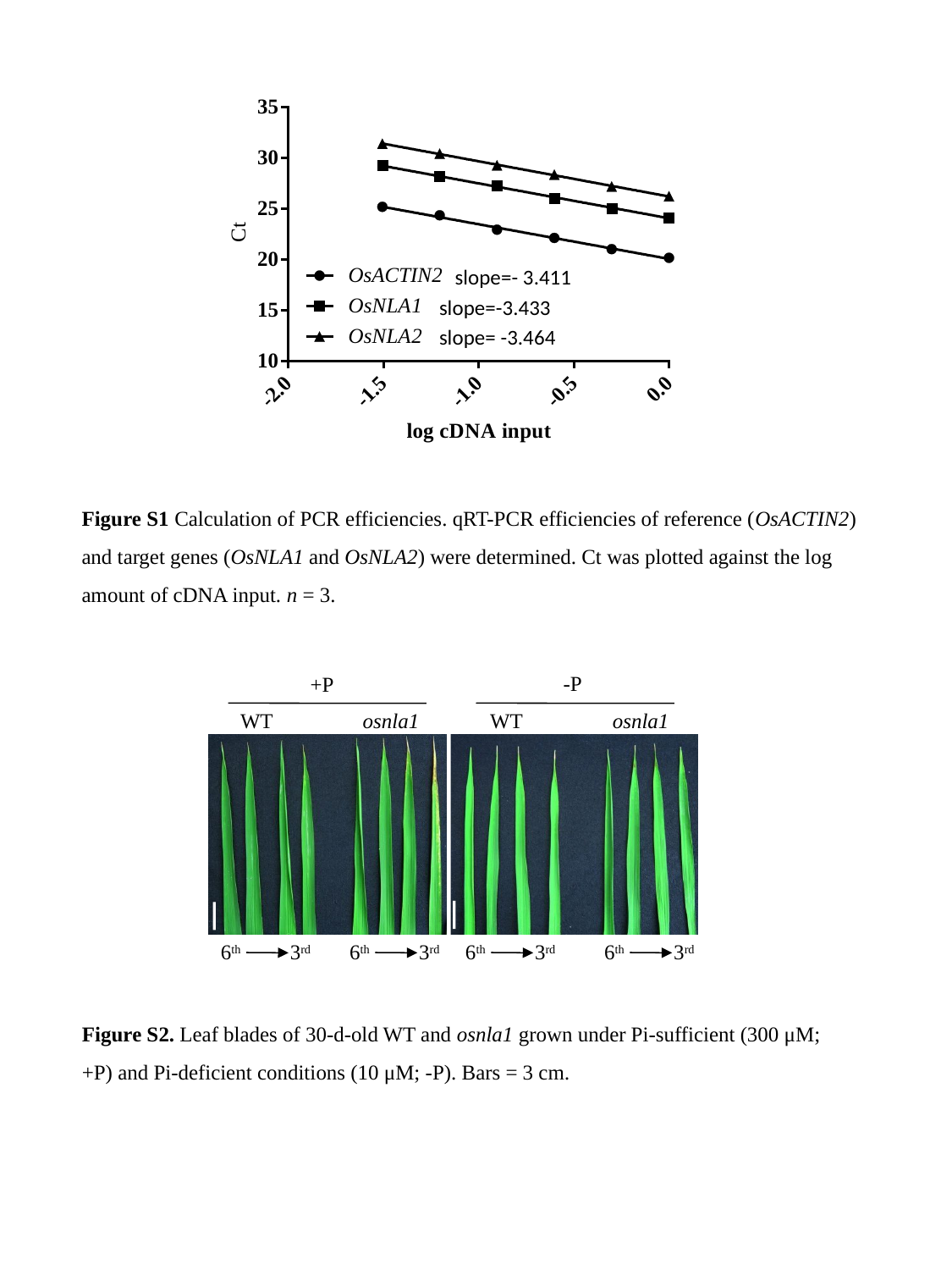

slope=- 3.411
slope=-3.433
slope= -3.464
Figure S1 Calculation of PCR efficiencies. qRT-PCR efficiencies of reference (OsACTIN2) and target genes (OsNLA1 and OsNLA2) were determined. Ct was plotted against the log amount of cDNA input. n = 3.
-P
+P
WT osnla1
WT osnla1
6th
3rd
6th
3rd
6th
3rd
6th
3rd
Figure S2. Leaf blades of 30-d-old WT and osnla1 grown under Pi-sufficient (300 μM; +P) and Pi-deficient conditions (10 μM; -P). Bars = 3 cm.
