# Supplementary material for: Characterization of the rice NLA family reveals a key role for OsNLA1 in phosphate homeostasis
Source: Rice (N Y). 2017 Dec 28;10:52. doi: 10.1186/s12284-017-0193-y (PMC5745205; doi:10.1186/s12284-017-0193-y)
Supplement: Supplementary file 3 — Primers used in this study. (DOCX 14 kb) [file 12284_2017_193_MOESM3_ESM.docx]

| Primers used in this study. |  |
| --- | --- |
| Primer for RT-PCR |  |
| OsNLA1-RT-F | GGAATTCAAAGCTCAAGCCCA |
| OsNLA1-RT-R | TCACATGCCCAAGAATGCCCT |
| OsACTIN2-RT-F:  OsACTIN2-RT-R: | CAACACCCCTGCTATGTACG  CATCACCAGAGTCCAACACAA |
| Primer for qRT-PCR |  |
| OsNLA1-QRT-F | ACATGTTCGATTTGCTTGGA |
| OsNLA1-QRT-R | CAGCAGAACAGCTGCACAAG |
| OsNLA2-QRT-F1 | AGGCGAAGTGTCCTGTATGC |
| OsNLA2-QRT-R1 | TCCAGTAATCCTTGCTCCTTGT |
| OsPT1-QRT-F | CATTGGTGTCATCTCTTTTCC |
| OsPT1-QRT-R | AGCATATCCATTGCATTCTTT |
| OsPT2-QRT-F | CACAAACTTCCTCGGTATGCT |
| OsPT2-QRT-R | GAAACCCCACAAATCCACAAC |
| OsPT4-QRT-F | CGTCATCGGGTTCTTCTTCAC |
| OsPT4-QRT-R | ACCAGAATAATCATCCATTGCACAT |
| OsPT6-QRT-F | GCCCCTGCAAACTGTACTG |
| OsPT6-QRT-R | AGCCAGGCCAGTTATATATCAAC |
| OsPT8-QRT-F | CCTACTTGTGTTTGTCTATGTG |
| OsPT8-QRT-R | GTGCCAAATTGCTGGTCTG |
| OsPT10-QRT-F | GAGCTCGCACCTCAGCAT |
| OsPT10-QRT-R | GAGTTCACTCACACGGAGACC |
| OsACTIN2-qRT-F | CAACACCCCTGCTATGTACG |
| OsACTIN2-qRT-R | CATCACCAGAGTCCAACACAA |
